# Tabanidae (Diptera) of Amazônia XXI. Descriptions of *Elephantotus* gen. n. and *E. tracuateuensis* sp. n. (Diachlorini) from the Brazilian coast

**DOI:** 10.3897/zookeys.395.7056

**Published:** 2014-04-01

**Authors:** Inocêncio de Sousa Gorayeb

**Affiliations:** 1 Museu Paraense Emílio Goeldi, Zoology, Entomology. Av. Perimetral No1901, Bairro Terra Firme, CEP 66117-070, Belém, Pará, Brazil

**Keywords:** Taxonomy, new genus, coastal horse flies, Amazon basin

## Abstract

*Elephantotus*, a new genus of Tabanidae from the Amazon coast, Brazil, is described based on a new species *E. tracuateuensis*. Five females were collected in Pará State, and a male in Maranhão State. Arguments are presented for separating the new genus from *Dasybasis*, as well as the possibility of its occurrence being related to the nesting sites of coastal birds. The new species is characterized by its large size (x = 2.15 cm, n = 5 females), glabrous eyes, reddish-brown tegument, light brown frontal callus not touching the edges of the eyes, extending up to the vertex that has traces of ocelli, basal plate of the antennal flagellum with obtuse angle, without a tooth or spine, wings hyaline, with brown basal cells, without appendix in the fork of vein R_4+5_, and genital furca wide with extended flaps.

## Introduction

Intensive collecting efforts along the Amazon coast during the last 15 years have enriched our knowledge of the fauna of the states of Amapá, Pará and Maranhão in Brazil, including the collection of five females and one male identified as Diachlorini – being described here as a new genus and species. The tribe Diachlorini has the largest number of genera and species within the family Tabanidae, comprising 36 genera with 594 species out of 1,205 described species of neotropical horse flies (Coscarón and Papavero 2009, Henriques et al. 2012). The most recent work with the tribe in that region was published by Henriques and Krolow (2013), describing the genus *Muscotabanus* and the species *Muscotabanus rafaeli* from the Brazilian Amazon.

## Materials and Methods

Specimens were collected using Malaise type flight-intercept traps (Gressit and Gressit 1962) that were installed at the edge of a mangrove forest in relatively open marsh land near a shorebird nesting site (including *Eudocimus ruber*, *Nycticorax nycticorax*, *Ardea alba*) (Schories and Gorayeb 2001), and with tubular fluorescent lamps (40 W) and 250 W tungsten filament and mercury vapor bulbs.

The specimens collected were deposited in the Museu Paraense Emílio Goeldi (MPEG) in Belém, Pará State, the Instituto Nacional de Pesquisas da Amazônia (INPA) in Manaus, Amazonas State, and the Centro de Estudos Superiores de Caxias (CESC/UEMA) at the Maranhão State University. Specimens and genitalia were examined, measured, and illustrated using a Zeiss Stemi SV 11 stereomicroscope with a coupled *camera lucida*, and photographed using a Leica M205 C microscope with a coupled Leica DFC 295 camera; Leica Application Suite (LAS V3.6) software was used to process the digital images.

The measurements given in the description of the holotype females are followed by parentheses with the ranges of the five specimens.

The terminology follows Cumming and Wood (2009) and Burger (2009).

## Results

### Elephantotus gen. n.

http://zoobank.org/B42B061E-C0FE-471D-8EF8-6B91E3CA6AEF

http://species-id.net/wiki/Elephantotus

#### Etymology.

Latin. *Elephantotus* = elephant. Masculine. Refers to the large sizes of the species and the shape of the furcation of the female genitalia, which have large side flaps – resembling an elephant’s head and ears in frontal view.

#### Type species.

*Elephantotus tracuateuensis* sp. n.

#### Diagnosis.

Eyes glabrous, solid-colored. Frons slightly divergent above. Frontal callus light brown, narrower than frons, extending to vestiges of ocelli on vertex. Basal plate of antennal flagellum with obtuse angle, without tooth or spine. Palpus approximately the same length as antenna. Labella completely membranous. Thorax with reddish-brown integument with irregular yellow spots, black median stripe and yellow stripes on sutures. Wing hyaline with brown basal cells, yellowish-brown veins, except basicostal which is brown, vein R_4+5_ fork without appendix. Genital furca wide, with flaps extended laterally. Goblets of genital ducts short.

In the key to the genera of Tabanidae Fairchild (1969), *Elephantotus* gen. n. proceeds to dichotomy 44 (p. 236) of *Dasybasis* Macquart. *Dasybasis* differs from *Elephantotus* by having a generally gray-colored body; eyes with sparse hairs; quadrangular frontal callus touching the eyes; ocellar triangle and ocelli vestigial; abdomen with longitudinal stripes and triangles on tergites (Coscarón and Philip 1967, González 1999). *Elephantotus tracuateuensis* sp. n. evidently does not belong to the genus *Dasybasis* which is characteristic of the neotropical southern temperate zone, with the exception of some species such as *Dasybasis montium* (Surcouf), 1919 and *Dasybasis schineri* (Kröber), 1931 occurring in Venezuela, Colombia and Ecuador (Coscarón and Papavero 2009).

The characteristics of some genera of Diachlorini (Fairchild, 1969) are presented here to differentiate them from *Elephantotus* gen. n.:

– *Catachlorops* Lutz – Tubercle on vertex distinguished and prominent, rarely rudimentary; proboscis usually with labella small, compact, completely or partially sclerotized, rarely completely membranous; antenna slender, dorsal spine slender, pointed, rarely shorter than basal plate; wing almost always with splotches, rarely faintly tinged, never completely hyaline.

– *Dasychela* Enderlein – not a strong or robust species, brown; wing spotted brown, hyaline or lighter colored at the end of basal cell and wing apex.

– *Phaeotabanus* Lutz – thorax without stripes; frontal callus small and round; labella much or completely sclerotized; wings usually with dark designs.

– *Stenotabanus* Lutz – most species small; eyes smooth with at least two transverse bands; frontal callus usually as wide as frons.

### Elephantotus tracuateuensis sp. n.

http://zoobank.org/DE6A409B-9C29-49A7-9212-49FA631BC247

http://species-id.net/wiki/Elephantotus_tracuateuensis

Figures 1 –17

#### Diagnosis.

Body 2.15 cm (n = 5 females), with reddish-brown integument. Eyes glabrous. Frons slightly divergent above. Frontal callus light-brown, narrower than frons, extending to vestiges of the ocelli. Antenna with scape and pedicel brown, with sparse pruinescence, flagellum orange with flagellomeres brown darkening to black at the last segment, basal plate orange, with obtuse angle. Thorax with reddish-brown integument with irregular yellow spots, black median stripe and sub-lateral yellow stripes on sutures. Wing hyaline, with brown basal cells and yellowish-brown veins, except basicostal which is brown, without appendix at forking of vein R_4+5_. Legs with glossy light-brown integument, covered with thin white pruinescence, hind legs darker. Abdomen with reddish-brown integument banded transversely with strips of yellow-brown integument in median and anterior regions of the segments. Genital furca wide, with flaps extending laterally. Goblets of genital ducts short.

#### Description of the female

(Figs 1–10).

**Figures 1–10.**
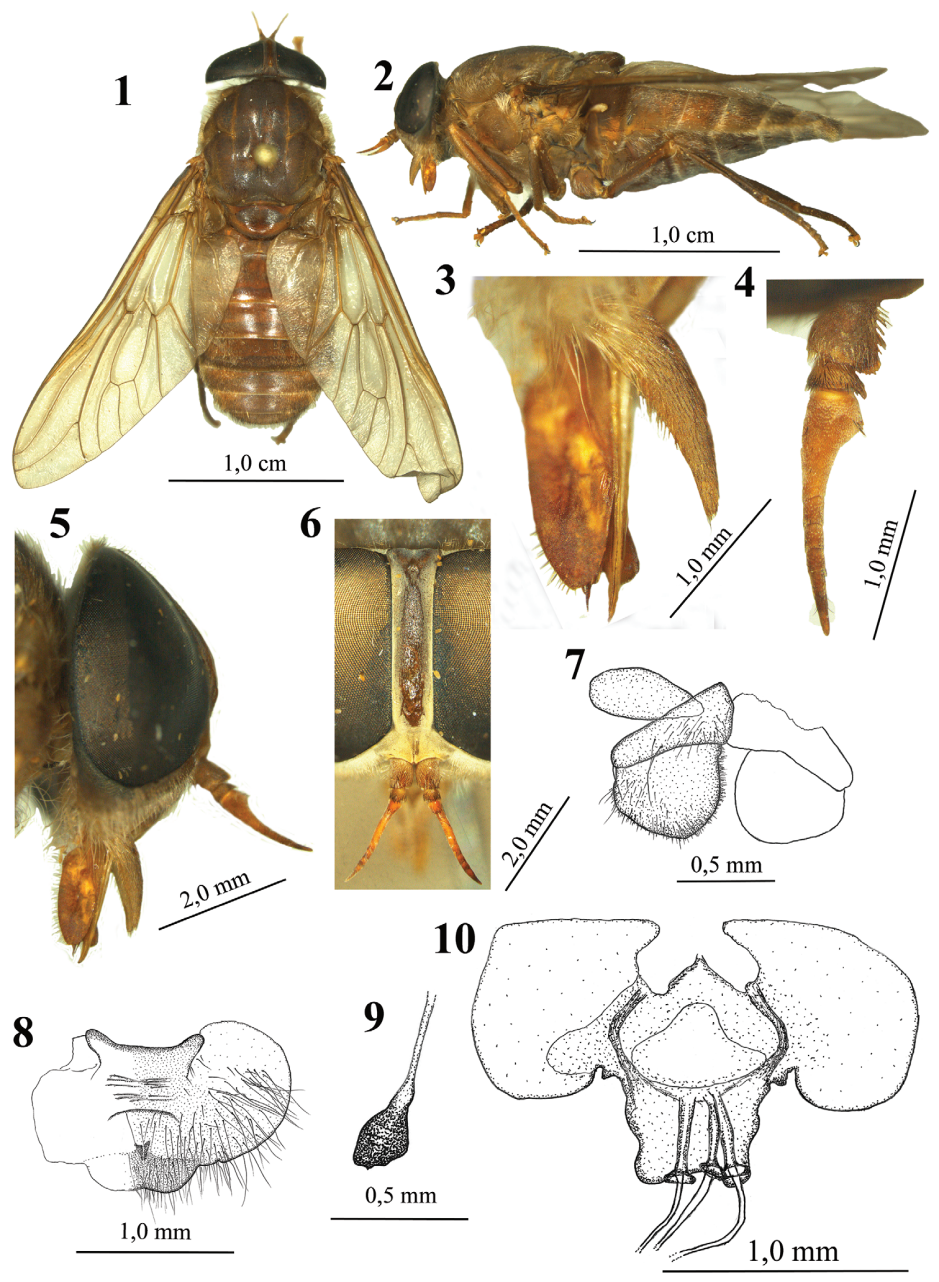
Female *Elephantotus tracuateuensis* sp. n.: **1** Body in dorsal view **2** Body in lateral view **3** Palpus, labella and stilets **4** Antenna **5** Head in lateral view **6** Frons **7** Tergites 9, 10 and cerci **8** Sternite 8 and gonapophyses **9** Spermatheca **10** Genital furca.

Body length 2.25 cm (1.99 to 2.27 cm), width of scutum at level of the transverse suture 7.5 mm (6.1 to 7.5 mm), wing length 2.15 cm (1.87 to 2.15 cm), wing width 6.7 mm (5.9 to 6.7 mm), height of frons 3.2 mm (2.6–3.2 mm), width of frons at vertex 0.8 mm (0.6–0.9 mm), width of frons at the base 0.7 mm (0.6–0.8 mm), frontal index 4.38 (3.90 to 4.38), divergent index 1.10 (1.02 to 1.11).

Head. Eyes glabrous. Frons (Fig. 6) slightly divergent above, swollen, yellow-whitish pruinescence; callus light-brown, set well away from margins, extending to vestiges of ocelli and vertex; setae white and yellow at apex. Subcallus covered with yellowish-white pruinescence. Antenna (Fig. 4) with scape brown, whitish-yellow pruinescence, black setae on upper lateral and dorsal areas, whitish-yellow setae on lower lateral and ventral areas; pedicel brown with less pruinescence, with black setae but with some scattered yellow setae on distal half; flagellum orange with brown to black flagellomeres on last segment; basal plate with obtuse angle, orange, with sparse whitish-yellow pruinescence, with black setae at corners, and four setae on distal lateral half; flagellomeres with black setae. Face, parafacialia, gena and postgena coated whitish-yellow pruinescence, and yellow setae. Palpus (Fig. 3) yellow, with yellow pruinescence, first segment with long yellow setae, second segment with black setae yellowish brown at base, inner side without setae. Proboscis orange with yellowish-white ventral bristles on basal half but black on ventral area of labella; labella large, approximately equal in size to the second palpus segment. Occiput with white pruinescence and yellow setae on dorsal edge.

Thorax. Scutum with reddish-brown integument with irregular yellow spots, black median stripe, and yellow stripes on sutures; pruinescence weakly gray-white when viewed at certain angles; short bristles bright yellow, orangish in posterior region of the scutum, longer white bristles in tuft on posterior areas of the supra-alar and post-alar lobes. Scutellum reddish-brown with anterior half dark brown, pruinescence grayish-white, with yellow and white setae on lateral sides. Pospronotal lobe whitish-yellow with white pruinescence and yellow setae. Notopleura yellow with pruinescence and setae yellow. Pleura and coxae with reddish-brown integument, coated with dense grayish-white pruinescence and yellowish-white setae. Legs with glossy light brown integument, covered with sparse white pruinescence, hind legs darker; femora with white setae; fore and median tibia with yellow-orange setae, brown setae scattered on the dorsum of distal quarter; posterior tibia with long brown setae, and short yellow-orange setae on ventral region; tarsi with brown to black setae on dorsum. Wing hyaline with brown basal cells (Fig. 1). Tegula brown, with yellow pruinescence and white setae. Veins yellowish-brown, except for brown costal vein; no appendix at fork of vein R_4+5_; vein M_3_ not reaching the wing margin. Calypter yellow, coated with white pruinescence, fringe of proximal calypter with brown bristles, distal calypter with white bristles. Subalar sclerite process brown on proximal half and yellow distally, coated with white pruinescence, with distal tuft of white setae. Halter brown, yellow distally coated with white pruinescence.

Abdomen. With reddish-brown integument, with transversal bands of yellow-brown integument on the median and anterior regions of the segments; coated with sparse grayish-white pruinescence and glossy white-yellowish setae, tergite 7 with brown setae.

Terminalia. Tergites 9, 10 and cerci as in Figure 10; tergite 9 small, tergite 10 subtriangular, cerci with relatively short setae for this family. Sternite 8 very wide, and gonapophyses as in Figure 8; spermatheca and genital furca as in Figures 9 and 10, with furca clearly very wide, with flaps extending laterally, goblets of genital ducts short; spermatheca club-like, relatively large as compared to other species.

Variations. Body pruinescence varies from white, to grayish-white, to yellow. The setae vary from white, to yellowish-white, to orange; also from matte to glossy. M_3_ veins vary, and do not reach the margins of either wing in the holotype, and only the right wing margin in the paratype male. The splotches at the base of wings vary from dark brown to hyaline. The integument is generally reddish-brown, but areas of the scutum, scutellum, tergites, and sternites have variable and irregular yellowish-brown stains.

#### Description of the male

(Figs 11–17).

**Figures 11–17.**
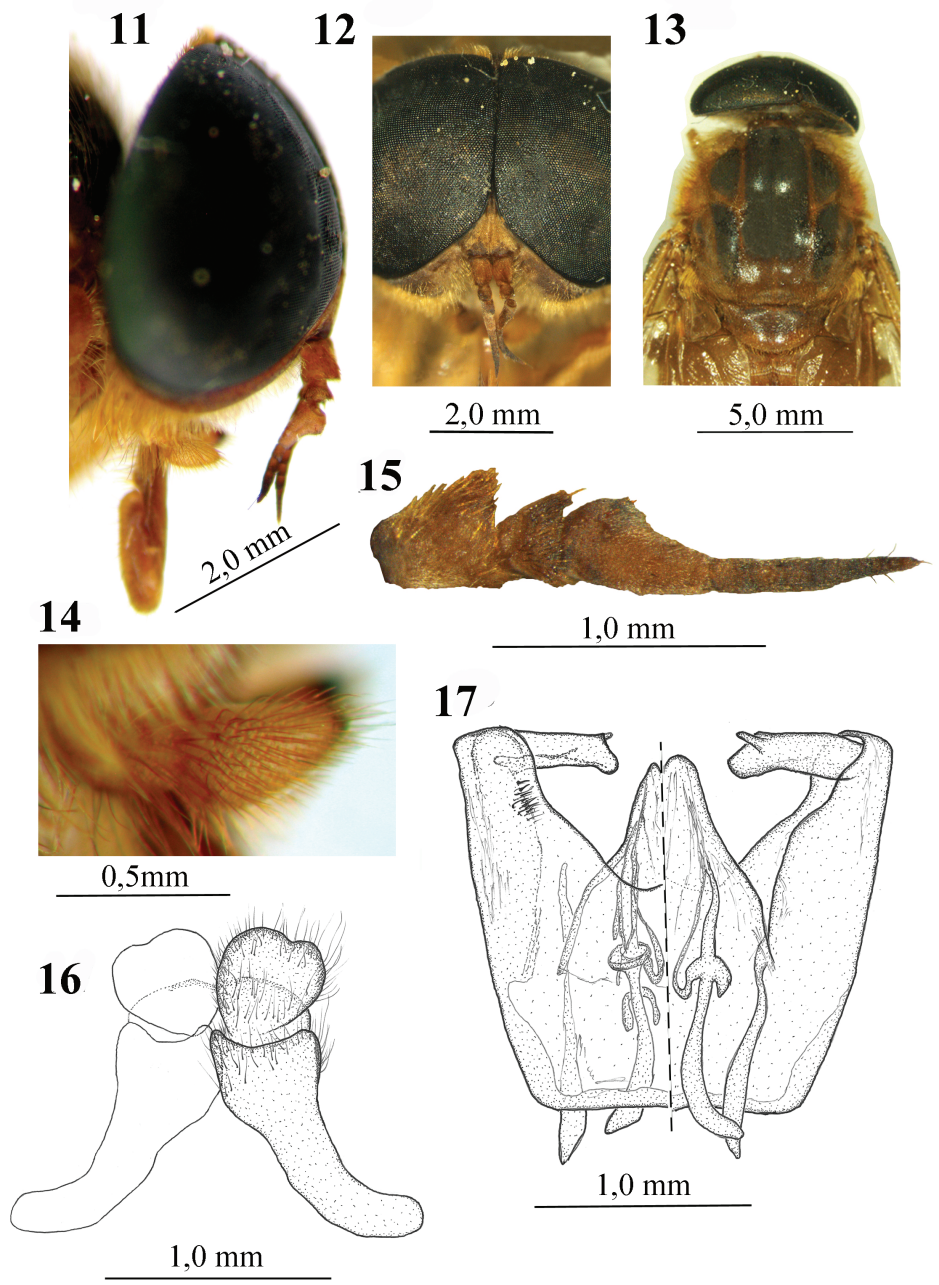
Male of *Elephantotus tracuateuensis* sp. n.: **11** Head in lateral view **12** Head in frontal view **13** Mesothorax and scutellum in dorsal view **14** Antenna **15** Palpus **16** Epandrium and cerci **17** Aedeagus.

Eyes with ommatidia of similar sizes. Smaller than female, length body 13.5 mm, wing length 12.6 mm, wing width 6.3 mm. Setae generally darker than those of the female, yellow-orange; no white setae. Palpus (Figs 11 and 14) with the first and second segments coated with pruinescence and long, yellow setae, the second slightly larger than the first. Integument usually with fewer spots, reddish brown and less intense than the female, with the exception of the scutum, which has well-defined black spots, as in Figure 13. Abdomen with brown setae on the median posterior areas of sternites. Hind tibia and tarsus without black setae, all of them orange. Wing with basicostal and costal cells yellow, vein M_3_ incomplete on right wing, not reaching the edge of the wing. Genitalia as in Figures 16 and 17; epandrium very long, and cerci heart-shaped with invagination on distal edge; aedeagus with dististilum with evident spine, and gonostilum with few setae on upper ventral face.

#### Distribution.

Brazil (states of Maranhão, Pará).

#### Etymology.

The specific epithet refers to the town of Tracuateua that belonged to the municipality of Bragança, on the coast of Pará State, Brazil.

#### Material examined.

Holotype ♀, BRAZIL, Pará, Bragança, Isla Canela, 0°47'06"S, 46°43'41"W, IX-27 to X-5-1995, Malaise trap, ocean side of the island, mangrove margin, Col.: N. Bittencourt (MPEG).

Paratypes. 1 ♀, Same as holotype (MPEG), 2 ♀ Idem (XI-8 to XI-13-1995, 1 ♀, MPEG, 1 ♀, INPA), 1 ♀ Brazil, Pará, Augusto Corrêa, Mandarité Beach, 0°52'38"S, 46°27'47"W, X-21-2001, fluorescent light, Cols.: L. A. Souza & I. S. Gorayeb (MPEG), 1 ♂, Brazil, Maranhão, São Luiz, Res. Itapiracó, Arm. Light (fluorescent lamps (40 W) and 250 W tungsten filament and mercury vapor bulbs), I-19 to I-20-2004, Cols.: J. T. Camara & J. W. P. Camara Jr. (CESC / UEMA).

## Discussion

I understand the concern that this genus is not based on a phylogenetic analysis of Diachlorini, but believe this genus to represent a unique lineage because it has the following characteristics not found in any other Diachlorini: large frontal callus, occupying more than half the frons, not touching the margins, slightly swollen; short proboscis with large membranous labella, the same size as the antennal flagellum; genital furca very wide, with flaps extended laterally; genital ducts to the goblets, short; and the following combined characters, glabrous eyes, wings hyaline without appendix on vein R_4+5_, basal plate antenna with obtuse angle without tooth.

Like other horse fly species that occur exclusively in coastal ecosystems, the specimens of *Elephantotus* gen. n. were collected along the coastline near an extensive nesting area of coastal birds. It probably evolved in association with large concentrations of warm-blooded animals, and we therefore suggest that other similar coastal areas be investigated for other species of horse flies that may have evolved in association with these flocks of birds.

## Supplementary Material

XML Treatment for Elephantotus

XML Treatment for Elephantotus tracuateuensis
